# World AIDS Day Commemoration

**DOI:** 10.1186/1742-6405-10-28

**Published:** 2013-12-13

**Authors:** Kailash Gupta

**Affiliations:** 1 Editor-in-Chief, AIDS Research and Therapy

## 

This Editorial is to commemorate “World AIDS Day” by increasing the awareness of AIDS, its treatment and prevention strategies.

Education is the single most crucial factor that can eradicate AIDS.

On the World AIDS Day, let us commit ourselves to learning and educating the world the scourge of AIDS.

What is World AIDS Day? (http://www.worldaidsday.org/about-world-aids-day.php)

World AIDS Day is held on 1 December each year and is an opportunity for people worldwide to unite in the fight against HIV, show their support for people living with HIV, and to commemorate people who have died. World AIDS Day was the first ever global health day and the first one was held in 1988.

Between 2011-2015, World AIDS Days will have the theme of “Getting to Zero: Zero new HIV infections. Zero discrimination. Zero AIDS related deaths”. The World AIDS Campaign focuses on “Zero AIDS related deaths” signifies a push towards greater access to treatment for all; a call for governments and pharmaceutical companies to act now. It is a call to honor promises like the Abuja declaration and for African governments to at least hit targets for domestic spending on health and HIV. (http://www.who.int/campaigns/aids-day/2013/en/)

Further Reading: http://en.wikipedia.org/wiki/World_AIDS_Day

As a tribute on the World AIDS Day *AIDS Research and Therapy* is publishing five high profile review articles to show our commitment in educating the world of the leading therapeutics and prevention efforts conducted by the dedicated research community. The articles span from Molecular Biology (Wilusz) to Humanities (Ramjee and Daniels) strategies. The goal has been to increase the awareness of therapeutics and suffering of the AIDS afflicted communities in post-HAART (highly active antiretroviral therapy) era. Specifically:

Dr. Wilusz presents persuasive arguments for novel potential therapeutics strategies that take into account the unique post-transcriptional events in HIV replication. For example, involvement of HIV TAT protein on highly structured 5’ end of HIV mRNAs and active repression of a promoter proximal signal for HIV polyadenylation.

Hsu et al. review serious non-AIDS events (SNAEs) such as malignancies, cardiovascular, renal and hepatic, neurological and bone disorders causing morbidity and mortality in HIV-infected individuals. Since SNAEs are multifactorial, the authors have suggested several novel strategies and interventions to reduce the burden of these disorders.

Similarly, the burden of metabolic complications (MC) in HAART treated individuals is high (de Paula et al.). The authors focus on the underlying mechanisms of MC highlighting pathophysiological and epidemiological aspects during HIV infection and during HAART treatment. Management of MC in HIV population is imperative.

The article by Achhra and Boyds reviews the clinical trials related to sparing antiretroviral treatment of the major class of nucleoside reverse transcriptase inhibitor (NRTI). The authors described the regimens currently used in NRTI-sparing regimen, their advantages and disadvantages, and the majority of clinical trials related to them.

Finally, but most importantly, sub-Saharan Africa bears the brunt of HIV infections accounting for almost two-thirds of world’s HIV infections (Ramjee and Daniels). The worst affected are younger women who have a larger prevalence of infection. Prevention strategies are crucial that take into consideration the socioeconomic and cultural aspects of women at large.

It is expected that these articles, on World AIDS Day, will increase the awareness of the AIDS burden and the strategies to combat and prevent it. Let the entire world learn the implications of AIDS so that it can be eliminated in a humanitarian way.

Kailash Gupta, Ph.D.

Editor-in-Chief

AIDS Research and Therapy

## Competing interests

The author declares that he has no competing interests.

